# Nuclear-specific accumulation of *telomerase reverse transcriptase* (*TERT*) mRNA in *TERT* promoter mutated follicular thyroid tumours visualised by in situ hybridisation: a possible clinical screening tool?

**DOI:** 10.1136/jclinpath-2021-207631

**Published:** 2021-05-19

**Authors:** L Samuel Hellgren, Ann Olsson, Ann Kaufeldt, Johan O Paulsson, Martin Hysek, Adam Stenman, Jan Zedenius, Catharina Larsson, Anders Höög, C Christofer Juhlin

**Affiliations:** 1 Department of Oncology–Pathology, Karolinska Institutet, Stockholm, Sweden; 2 Department of Pathology and Cytology, Karolinska University Hospital, Stockholm, Sweden; 3 Department of Molecular Medicine and Surgery, Karolinska Institutet, Stockholm, Sweden; 4 Department of Breast, Endocrine Tumors and Sarcoma, Karolinska University Hospital, Stockholm, Sweden

**Keywords:** in situ hybridization, thyroid neoplasms, pathology, molecular

## Abstract

**Aims:**

Upregulation of the *telomerase reverse transcriptase* (*TERT*) gene is a frequent finding in follicular thyroid carcinomas (FTCs) with metastatic features. The augmented expression is usually caused by *TERT* promoter mutations. As TERT protein immunohistochemistry might not correlate to *TERT* mRNA levels in follicular thyroid tumours, we therefore sought to determine if visualisation of *TERT* mRNA through in situ hybridisation could highlight high-risk cases.

**Methods:**

We collected formalin-fixated paraffin-embedded tissues from 26 follicular thyroid tumours; 7 FTCs, 2 follicular thyroid tumours of uncertain malignant potential (FT-UMPs) and a single Hürthle cell carcinoma with established *TERT* promoter mutations and gene expression, as well as 16 FTCs with no *TERT* gene aberrancy or gene expression, and assessed them using RNA Scope in situ hybridisation (ISH) and *TERT* probes targeting the two main *TERT* transcripts (*TERT1 and TERT2*).

**Results:**

*TERT 1* and/or *2* mRNA was found by ISH in 8/10 cases with established promoter mutations and mRNA expression, whereas all 16 cases without *TERT* gene aberrancies or gene expression were negative (Fisher’s exact p<0.001). Strikingly, *TERT* mRNA was visualised in the nuclear compartment only, thereby corroborating earlier studies suggesting a non-conventional role for *TERT* in tumour biology. Moreover, *TERT* mRNA expression was scattered across the tissue sections and only found in a few percentages of tumour nuclei.

**Conclusions:**

*TERT* mRNA seems to be focally expressed and localised exclusively to the nucleus in *TERT* promoter mutated follicular thyroid tumours, possibly reflecting a true biological and unorthodox phenomenon worthy of further investigations.

## Introduction

In thyroid cancer, prognostication and treatment-based decisions are largely dictated by the tumour node metastasis (TNM) staging system.^1 2^ This classification model is based on tumour size and the presence of extrathyroidal extension, two parameters that are easy to retrieve and usually reproducible between different centres.^1^ However, there are instances in which these parameters are not sufficient to predict the outcome of the individual patient, and researchers have therefore sought additional prognostic markers to aid in this aspect. Mutations in the promoter region of the *telomerase reverse transcriptase* (*TERT*) gene are found in approximately 10%–15% of papillary thyroid carcinomas (PTCs) and follicular thyroid carcinomas (FTCs), and particularly poor-prognosis cases that are associated with adverse clinical features.^3–10^ Moreover, *TERT* promoter mutations are recurrently observed in more aggressive tumour types, such as poorly differentiated thyroid carcinomas (PDTCs) and anaplastic thyroid carcinomas (ATCs), thereby solidifying the relationship between this genetic aberration and worse clinical outcome in thyroid cancer.^11 12^ These mutations might also be able to pinpoint metastatic potential in histologically equivocal ‘follicular thyroid tumours of uncertain malignant potential’ (FT-UMPs).^13^

To date, there are two established hotspot *TERT* promoter mutations (denoted C228T and C250T) which are closely positioned and hence both interrogated by Sanger sequencing using a single primer pair.^14 15^ The mutations are thought to increase *TERT* gene transcription through the intensified recruitment of various transcription factors, and as *TERT* encodes the catalytic subunit of telomerase, this mechanism is thought to stimulate telomere lengthening and thus counteracts senescence-induced apoptosis.^7 16^ In thyroid tumours in general, *TERT* gene expression is invariably associated to malignant disease and worse clinical outcomes, and therefore constitutes a potential diagnostic and prognostic marker.^17–22^ In FTCs and FT-UMPs, other genetic mechanisms besides promoter mutations also seem to stimulate *TERT* mRNA expression, such as aberrant promoter methylation and copy number gain of the *TERT* gene locus.^9^ As the presence of *TERT* mRNA could constitute a marker of worse prognosis seemingly irrespectively of the causative genetic mechanism, we previously investigated whether or not TERT protein expression could constitute a surrogate marker for *TERT* gene activation in follicular thyroid tumours.^23^ However, we found no correlation between *TERT* mRNA levels and TERT immunoreactivity, and to our surprise, nuclear expression of *TERT* was largely absent. Given the known association between *TERT* gene output and worse clinical outcomes, we therefore turned our attention to in situ hybridisation (ISH). To our knowledge, this methodology has not previously been attempted in terms of visualising *TERT* mRNA in formalin-fixated paraffin-embedded (FFPE) follicular thyroid tumour specimen with known *TERT* promoter genotypes. The potential benefits of ISH include the possibility to detect *TERT* mRNA expression in clinical routine processed samples, as well as to visualise the specific *TERT* expressing cell type. As several reports indicate that benign thyroid lesions with concomitant thyroiditis may exhibit *TERT* mRNA due to constitutive expression in lymphocytes, ISH could in theory be a way to eliminate these false positives from a screening perspective.^19 24 25^

## Material and methods

### Study cohort

The study cohort was retrospectively collected, and consisted of 26 thyroid tumours previously characterised for *TERT* mRNA expression, *TERT* promoter mutations, *TERT* promoter methylation levels and *TERT* gene copy number.^9^ Baseline clinical, histopathological and molecular attributes are summarised in table 1. All patients were surgically treated at the Karolinska University Hospital, Stockholm, Sweden between 2013 and 2016, and all tumours were diagnosed using the most recent WHO criteria.^26^ A total of 7 FTCs, 2 FT-UMPs and a single Hürthle cell carcinoma with established *TERT* promoter mutations (based on Sanger sequencing results) and *TERT* mRNA expression (determined by quantitative real-time PCR; qRT-PCR) were included (cases 1–10), as were 16 FTCs absent of *TERT* promoter hotspot mutations and *TERT* mRNA expression (cases 11–26). None of the cases exhibited histological evidence of thyroiditis. A single de-identified multinodular goitre sample was included as a non-tumour reference.

**Table 1:** Summarised histopathological and clinical information

***TERT*** **promoter mutated and** ***TERT*** **mRNA expressing cases (n=10)**	
Median age at surgery (range)	71.5 (38–91)
Sex, female:male	6:4
Median tumour size in mm (range)	45 (15–100)
Histological type	wiFTC (n=6), FT-UMP (n=2), miFTC (n=1), HCC (n=1)
Median Ki-67 index (range)	7.5 (1.3–12)
Distant metastases/local recurrences	Lung met (n=2), bone met (n=2), local recurrence (n=1)
Average age of tissue blocks	2248 days
***TERT* promoter wildtype and *TERT* mRNA negative cases (n=16**)	
Median age at surgery (range)	50 (11–71)
Sex, female:male ratio	15:1
Median tumour size in mm (range)	42 (20–58)
Histological type	wiFTC (n=9), eaiFTC (n=2), miFTC (n=5)
Median Ki-67 index (range)	4.25 (1–12.5)
Distant metastases/local recurrences	None reported
Average age of tissue blocks	2234 days

eaiFTC, encapsulated angioinvasive follicular thyroid carcinoma; FT-UMP, follicular thyroid tumour of uncertain malignant potential; HCC, Hürthle cell carcinoma; miFTC, minimally invasive follicular thyroid carcinoma; wiFTC, widely invasive follicular thyroid carcinoma.

### In situ hybridisation

The ISH methodology was carried out using the RNAscope technology (Advanced Cell Diagnostics, CA, USA).^27^ We used two *TERT* probes: TERT1; RNAscope Hs-TERT, product no. 605511, targeting homo sapiens *telomerase reverse transcriptase* (*TERT*) mRNA transcript variant 1, and *TERT2*; RNAscope Hs-TERT-O2, product no. 494561, targeting homo sapiens *telomerase reverse transcriptase* (*TERT*) mRNA transcript variant 2 (Advanced Cell Diagnostics). As controls, we used a probe against the housekeeping gene *Peptidylprolyl Isomerase B* (*PPIB*) (product no. 313901) as positive control and the bacterial RNA sequence *Dihydrodipicolinate Reductase* (*dapB*) (product no. 310043) (Advanced Cell Diagnostics) as negative control. Validation of the methodology was performed using two serially sectioned FTC cases with previously established *TERT* promoter mutations and *TERT* mRNA expression as well as using mounted HeLa cells (ACD, product no. 310045) using both *TERT* and control probes (Advanced Cell Diagnostics). All probes were assessed using different methods for upholding the temperature during the pretreatment phase (pressure cooker vs water bath), and optimal signals were retrieved using the RNAscope Target Retrieval Standard for 15 min at 95°C, followed by protease treatment for 30 min. Slides were then processed according to a standardised protocol provided by the manufacturer. The hybridisation was performed using an ACD HybEZ II Hybridization System (Advanced Cell Diagnostics). Diaminobenzidine (DAB) was used as chromogen, and slides were counterstained in hematoxylin.

### Visualisation and scoring procedure

All slides were evaluated by an endocrine pathologist (unaware of the previous *TERT* gene screening outcomes) at ×400 magnification using a BX46 Olympus light microscope (Olympus, Tokyo, Japan). Images were captured using a ToupCam Industrial Digital Camera and the ImageView software. Both the cytoplasmic and nuclear compartments were analysed in all cases, and cases were noted as positive if distinct signals were envisioned in subsets of tumour cells. The entire slide was examined in ×400 magnification in order to visualise focal signals, and verified using ×1000 magnification. Negative cases were annotated as such if there was no clear-cut signal in any tumour cells.

### Statistical analyses

The statistical analyses (Fisher’s exact test, Mann-Whitney U) were performed using IBM SPSS Statistics V.27. P values<0.05 were considered significant.

## Results

### Control experiments

**Figure 1:**
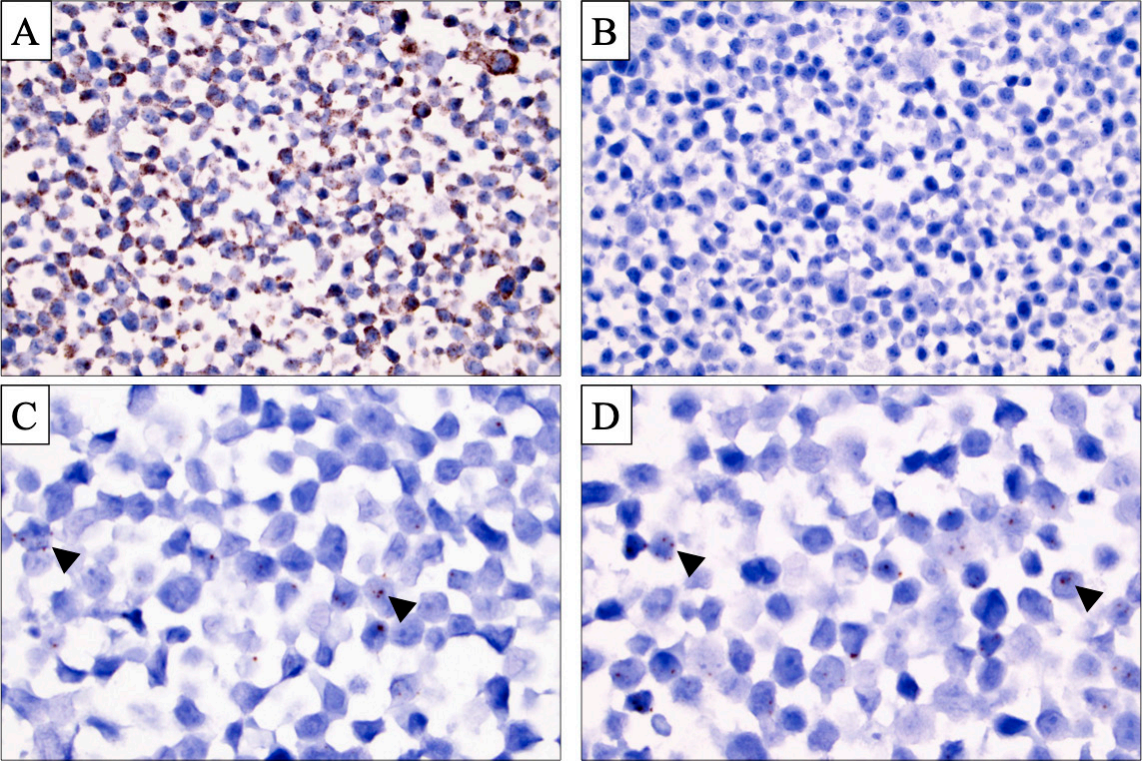
Control and *TERT* in situ hybridisation (ISH) signals in HeLa cells. (A) *Peptidylprolyl Isomerase B* (*PPIB*) housekeeping gene ISH displaying a strong, predominant cytoplasmic signal. Magnification ×400. (B) ISH probe directed at bacterial *Dihydrodipicolinate Reductase* (*dapB*) RNA displaying absent signals, serving as a negative control of the methodology. Magnification ×400. (C, D) *TERT1* (C) and *TERT2* (D) ISH at ×1000 magnification visualising subsets of HeLa cell with intense nuclear, dot-like signals. Black arrowheads highlight a subset of these signals.

Outcomes of the control experiments are detailed in figure 1. Using mounted HeLa cells (immortalised cervical cancer cell line known to express telomerase), strong and diffuse cytoplasmic housekeeping gene expression was evident, whereas negative controls (bacterial RNA sequence) were devoid of signals.^28^ Using *TERT1* and *TERT2* probes, a distinct, dot-like nuclear signal was evident in subsets of the HeLa cells. The cytoplasm of the HeLa cells was not stained. For subsequent analyses of the follicular thyroid tumours, a positive housekeeping control was included for each case, as well as a negative control in each experimental run. All follicular thyroid tumours and the multinodular goitre case stained positive for the housekeeping gene with abundant cytoplasmic expression, while consistently negative for the bacterial RNA sequence. The multinodular goitre sample was absent of signals using both *TERT* probes.

### TERT mRNA visualisation in follicular thyroid tumors using ISH

**Figure 2:**
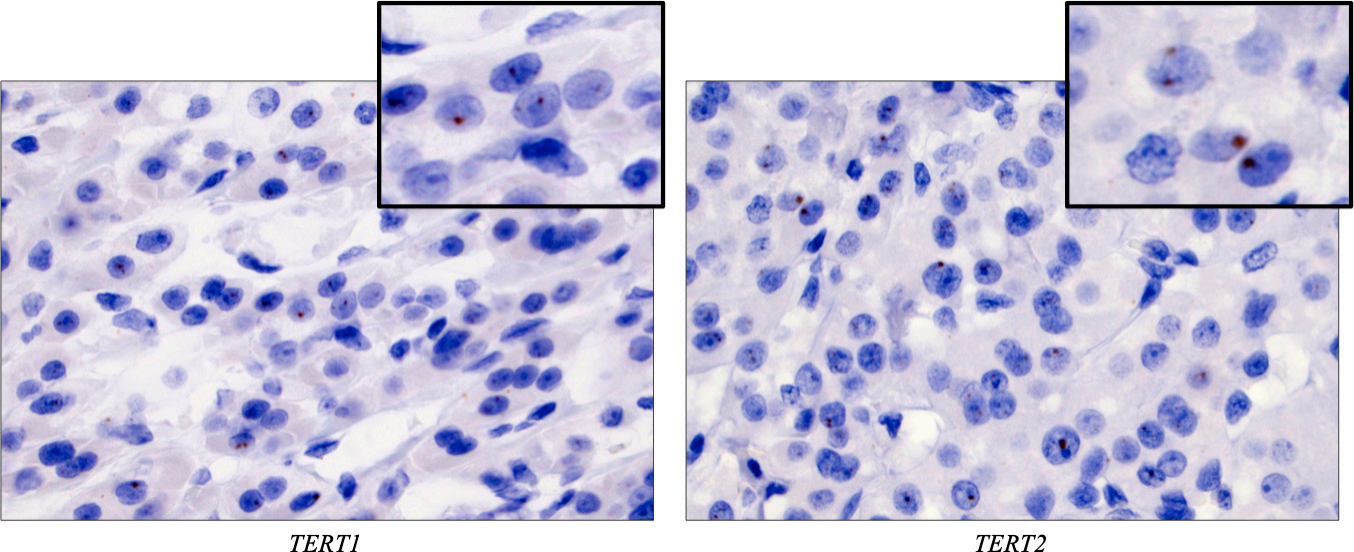
Focal, dot-like nuclear signals using both TERT in situ hybridisation probes were seen in the majority of *TERT* promoter mutated thyroid tumours with previously established *TERT* mRNA expression, represented here by case 10, a Hürthle cell carcinoma. Magnification ×400, with insets magnified ×1000.

**Figure 3:**
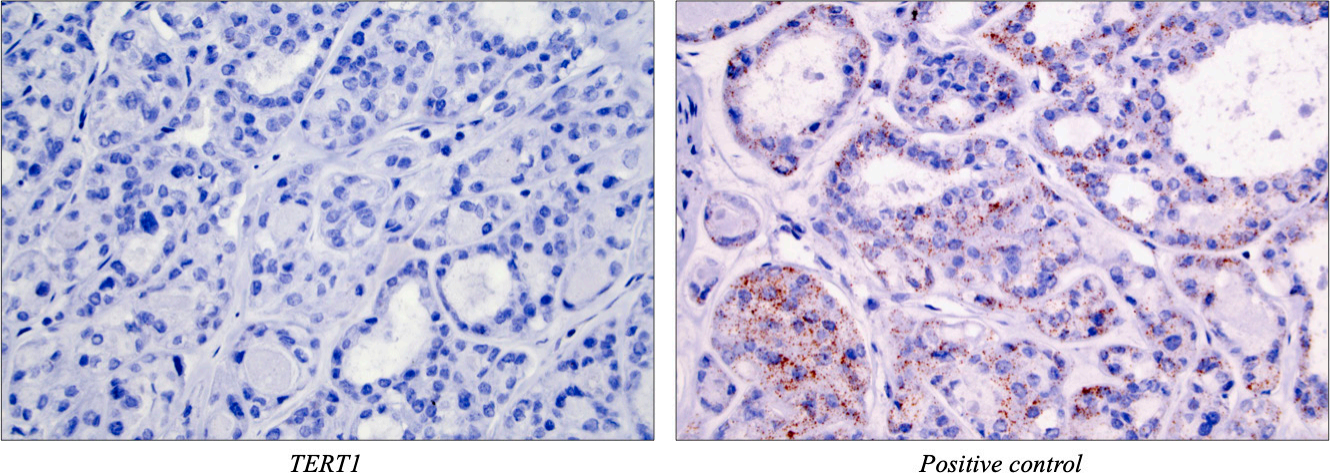
All follicular thyroid carcinomas (FTCs) without *TERT* promoter mutations and devoid of *TERT* mRNA as previously interrogated by quantitative real-time PCR were negative for in situ hybridisation signals using both *TERT1* and *TERT2* probes. Shown here is case 26, an encapsulated angioinvasive FTC, with a negative *TERT1* signal (left) while showing a clear cytoplasmic signal using the positive control (right). Magnification ×400.

The ISH staining outcomes are detailed in table 2 and illustrated in figures 2 and 3. The majority of the 10 thyroid tumours with established *TERT* promoter mutations and *TERT* mRNA expression demonstrated nuclear, dot-like *TERT* ISH signals in subsets of tumour cells (*TERT1*; n=6/10; 60%, *TERT2*; n=7/10; 70%) (figure 2). Cytoplasmic signals were not seen in any case. Two cases were negative using both *TERT* probes. In five cases, focal positive nuclear signals were retrieved using both *TERT* probes, whereas the remaining three positive samples were only identified using one of the probes (*TERT1* in one case, *TERT2* in two cases). Both FT-UMPs with *TERT* promoter mutations displayed absent signals using the *TERT1* probe, but exhibited positive nuclear signals using *TERT2*. There was no apparent correlation between the level of relative *TERT* mRNA expression from previous qRT-PCR analyses and ISH outcome (data not shown). All 16 FTCs lacking *TERT* promoter mutations and *TERT* mRNA expression were devoid of ISH signals using both *TERT* probes (figure 3), although four cases displayed various amounts of DAB precipitation, making the scrutinising of these slides somewhat burdensome (data not shown). The tumour stroma (endothelial cells and fibroblasts) was consistently negative for *TERT* signals in all cases.

**Table 2:** *TERT* in situ hybridisation results

Sample number	Diagnosis	*TERT* promoter*	*TERT* expression (qRT-PCR)*	In situ hybridisation	
				*TERT1*	*TERT2*
1	wiFTC	Mutated	Yes	Focal	Neg
2	wiFTC	Mutated	Yes	Focal	Focal
3	wiFTC	Mutated	Yes	Neg	Neg
4	wiFTC	Mutated	Yes	Focal	Focal
5	wiFTC	Mutated	Yes	Focal	Focal
6	miFTC	Mutated	Yes	Focal	Focal
7	wiFTC	Mutated	Yes	Neg	Neg
8	FT-UMP	Mutated	Yes	Neg	Focal
9	FT-UMP	Mutated	Yes	Neg	Focal
10	HCC	Mutated	Yes	Focal	Focal
11	miFTC	Wildtype	None	Neg	Neg
12	miFTC	Wildtype	None	Neg	Neg
13	eaiFTC	Wildtype	None	Neg	Neg
14	miFTC	Wildtype	None	Neg	Neg
15	wiFTC	Wildtype	None	Neg	Neg
16	wiFTC	Wildtype	None	Neg	Neg
17	miFTC	Wildtype	None	Neg	Neg
18	wiFTC	Wildtype	None	Neg	Neg
19	wiFTC	Wildtype	None	Neg	Neg
20	miFTC	Wildtype	None	Neg	Neg
21	wiFTC	Wildtype	None	Neg	Neg
22	wiFTC	Wildtype	None	Neg	Neg
23	wiFTC	Wildtype	None	Neg	Neg
24	wiFTC	Wildtype	None	Neg	Neg
25	wiFTC	Wildtype	None	Neg	Neg
26	eaiFTC	Wildtype	None	Neg	Neg

*Retrieved from Paulsson *et al.* 9

eaiFTC, encapsulated angioinvasive follicular thyroid carcinoma; FT-UMP, follicular thyroid tumour of uncertain malignant potential; HCC, Hürthle cell carcinoma; miFTC, minimally invasive follicular thyroid carcinoma; Neg, negative; qRT-PCR, quantitative real-time PCR; wiFTC, widely invasive follicular thyroid carcinoma.

The sensitivity and specificity for *TERT* ISH to detect cases with underlying *TERT* promoter mutations and *TERT* mRNA expression was 60% and 100%, respectively, for *TERT1* and 70% and 100%, respectively, for *TERT2*. Accepting positive signals with any of the two probes, the sensitivity rose to 80%. The positive predictive value was 100% for either probe, whereas the negative predictive values were 80% (*TERT1*), 84% (*TERT2*) and 89% (any of the two probes). There was a strong correlation between the visualisation of *TERT* mRNA expression using ISH and underlying *TERT* promoter mutations as well as evident *TERT* mRNA expression as interrogated via qRT-PCR (Fisher’s exact p<0.001). Moreover, there was no significant difference in the age of the tissue blocks selected for ISH analyses between mutation-positive (2248 days) and mutation-negative groups (2234 days) (Mann-Whitney U, p=0.75) (table 1). Also, the two tumours with established *TERT* promoter mutations and *TERT* mRNA expression that were negative on ISH using both *TERT* probes were not the two oldest cases among the mutation-positive tumours (data not shown).

## Discussion

*TERT* gene expression is invariably associated with poorer patient outcomes in thyroid cancer, and this dysregulation has in turn been coupled to underlying *TERT* promoter mutations as well as alternate genetic mechanisms leading to increased *TERT* gene output.^3–5 7 9^ As *TERT* mRNA expression correlates poorly to TERT protein expression in follicular thyroid tumours, there are potential clinical benefits to develop a method that will correctly pinpoint *TERT* mRNA expression in clinical samples.^23^ Although qRT-PCR could be considered in this aspect, recent improvements of the ISH technique have made this method attractive for clinical purposes.^27^ First, not all institutions have the ability to collect and analyse fresh-frozen tissues, and second, *TERT* mRNA expression is recurrently reported in lymphocytes.^19 25 29^ Visualisation of *TERT* by ISH is performed on clinical routine FFPE material, and also enables the pathologist to detect spatial and tissue-specific distribution patterns that qRT-PCR cannot.

In our series, *TERT* signals were exclusively found in the nuclear compartment. This was true for the thyroid tumours as well as for the HeLa cells used as controls, suggesting a true biological role for nuclear *TERT* mRNA. Previous observations support our findings, in which *TERT* mRNA seems to aggregate in the nucleus when analysing various cancer cell lines as well as malignant melanomas.^30 31^ As we could not detect a nuclear signal in the negative controls, we do not suspect the findings of nuclear-specific *TERT* to be a false-positive observation. Moreover, as all cases exhibited a strong cytoplasmic housekeeping gene signal, this implies that RNA levels were intact even after 24–48 hours of formalin fixation. Therefore, the absence of cytoplasmic *TERT* signals is most likely not a consequence of poor RNA quality, but rather implies a biological phenomenon worthy of attention. Indeed, *TERT* mRNA molecules could potentially exhibit non-conventional roles besides acting as a ribosomal template for translation.

Interestingly, *TERT* ISH exhibited perfect specificity, as all *TERT* mRNA devoid FTCs were negative on ISH using both probes. Therefore, the method could potentially be of value for clinical screening purposes. In theory, the detection of *TERT* signals in a histologically confirmed FTC could therefore imply an underlying *TERT* gene aberrancy, which in turn is strongly associated with worse patient outcome.^9^ Moreover, studies using preoperative fine-needle aspiration biopsy material could also be valuable, as the expression of *TERT* could imply a clinically more burdensome tumour in need of more extensive interventions. However, *TERT*-positive cases only exhibited nuclear signals in small subsets of tumour cells, thereby forcing the pathologist to scrutinise the whole slide using high power (×400 or ×1000) magnification. This could be time-consuming from a clinical perspective, conferring a risk of failing to detect the area of positivity and misclassifying the tumour as negative. Indeed, our experience herein suggests that *TERT*-positive cells aggregate in small clusters and might be hard to detect if not the entire slide is investigated carefully. In a way, our findings thus bear similarities to current PD-L1 immunohistochemical scoring algorithms, in which very low cut-offs for positivity have been recommended, and several heterogeneous expression patterns have been reported.^32^ As of this, modern pathologists are thus getting acquainted to scoring principles based on regional and variable intensity across a tissue slide, and not only diffuse and unequivocal expression patterns. Indeed, the previous findings of *TERT* promoter mutational spatial heterogeneity in follicular thyroid tumours adds to the complexity of *TERT* visualisation for clinical purposes, and future studies will possibly need to address the potential benefit of multi-section analyses in highlighting focal *TERT* dysregulation.^33 34^

**Take home messages**

- *TERT* promoter mutations and *TERT* mRNA expression are prognostic markers of relevance in follicular thyroid tumours.
- *TERT* in situ hybridisation correctly pinpoints *TERT* aberrancies in the majority of cases by visualising exclusive nuclear signals.
- The methodology could be of potential value for clinical screening purposes and might imply unconventional roles for nuclear *TERT* mRNA in thyroid cancer development.

## Data Availability

All data relevant to the study are included in the article. The authors confirm that the data supporting the findings of this study are available within the article.
